# Ultrasound-guided distal versus conventional radial artery catheterization in older surgical patients: protocol for a randomized controlled noninferiority trial

**DOI:** 10.3389/fmed.2026.1985261

**Published:** 2026-10-19

**Authors:** Long Zhang, Shunli Diao, Liangguang Zhang, Xiaohua Li

**Affiliations:** 1 Department of Anesthesiology, Ningbo No. 6 Hospital, Ningbo, Zhejiang, China; 2 Ningbo Clinical Research Center for Orthopedics, Sports Medicine & Rehabilitation, Ningbo, Zhejiang, China

**Keywords:** arterial catheterization, distal radial artery, invasive blood pressure monitoring, noninferiority trial, older patients

## Abstract

**Background:**

Invasive radial arterial monitoring is frequently required in older surgical patients, but age-related vascular changes can make catheterization difficult. Distal radial access may improve catheter stability during wrist movement, shorten hemostasis, and preserve the conventional forearm segment; however, its smaller caliber may reduce technical success. This trial will determine whether ultrasound-guided distal radial artery catheterization is noninferior to conventional radial artery catheterization for first-attempt success in patients aged 65 years or older.

**Materials and methods:**

This single-center, parallel-group, randomized noninferiority trial will enroll 198 surgical patients aged 65 years or older who require invasive arterial pressure monitoring. Participants will be allocated 1:1 to distal or conventional radial artery catheterization. Both groups will receive the same real-time ultrasound-guided dynamic needle-tip positioning technique and a 20G*45 mm catheter; only the access site and limb position will differ. The operator cannot be masked, whereas postoperative outcome assessors and the statistician will be masked where feasible. The primary outcome is first-attempt success, defined as catheter placement with free aspiration and an undamped arterial waveform after one skin puncture. Secondary outcomes are overall assigned-site success, procedural efficiency, intraoperative catheter performance, access-site safety and hemostasis, and Doppler-confirmed arterial patency. Noninferiority will be concluded if the lower limit of the two-sided 95% confidence interval for the risk difference (distal minus conventional) is greater than −15 percentage points in both the intention-to-treat (ITT) and per-protocol (PP) populations.

**Ethics and dissemination:**

The protocol was prepared in accordance with SPIRIT 2025. This research protocol was approved by the Ethics Committee of Ningbo No. 6 Hospital on 12th March 2026 (Approval No.: 2026—10L), and written informed consent will be obtained before randomization. Results will be disseminated through peer-reviewed publications and scientific presentations. De-identified data and analysis code will be shared after publication, subject to ethics approval and privacy requirements.

**Clinical Trial Registration:**

www.chictr.org.cn; identifier ChiCTR2600124338.

## Highlights

- The trial focuses specifically on patients aged 65 years or older, an anatomically and clinically relevant population that remains underrepresented in previous comparative trials.
- Both groups will receive an identical, standardized real-time ultrasound-guided technique, thereby isolating the effect of the access site from the effect of the imaging modality or needle-guidance technique.
- Noninferiority will be assessed in both the ITT and PP populations, with objective procedural definitions and masked postoperative assessment where feasible.
- Duplex assessment of arterial patency and standardized recording of waveform instability will extend evaluation beyond immediate cannulation success.
- The single-center setting, unavoidable lack of operator masking, short follow-up, and operator dependence may limit generalizability and prevent definitive assessment of rare or delayed vascular complications.

## Introduction

Invasive arterial catheterization is widely used for beat-to-beat blood pressure monitoring and repeated arterial blood sampling during surgery (1, 2). The conventional radial artery at the volar wrist is the preferred site because it is superficial, accessible, and usually supported by collateral hand circulation (3). Catheterization can nevertheless be difficult in older adults because arterial calcification, reduced compliance, tortuosity, atherosclerotic change, and weak pulsation are common and may be compounded by diabetes, hypertension, peripheral vascular disease, or low-flow states. Repeated attempts increase pain and tissue injury and may lead to hematoma, vasospasm, or arterial occlusion (4, 5). Although ultrasound guidance improves first-attempt success and reduces attempts and hematomas in older patients (6, 7), it does not resolve which radial access site is preferable.

The distal radial artery in the anatomical snuffbox or dorsoradial hand has been proposed as an alternative to the conventional wrist site. Its position over a firm osseous base may facilitate compression, reduce catheter kinking or waveform damping during wrist movement, and preserve the conventional forearm radial artery for future access. These advantages may be relevant during prolonged surgery or when operative positioning requires wrist movement. However, the distal artery is generally smaller and may be deeper, potentially reducing first-attempt success and prolonging catheterization. The clinical value of distal access, therefore, depends on whether its potential gains in catheter stability and access-site safety offset any loss of procedural efficiency.

Comparative evidence remains inconsistent. Xiong et al. reported noninferior first-attempt success with distal versus conventional access (87.7% vs. 91.3%), together with fewer dampened waveforms but a longer procedure (8). Maitra et al., however, found lower first-attempt success with distal access (57% vs. 77%) and longer catheterization time (9). More recent trials in critically ill and anesthetized adults found comparable insertion success but fewer waveform-loss events with distal placement, particularly during wrist movement (10, 11). This research group has also reported successful cases (12). A 2026 meta-analysis of 12 randomized trials involving 1,790 participants found no overall difference in first-attempt success and suggested fewer hematomas, bleeding events, catheter blockages, and unstable waveforms with distal access, although insertion took longer (13). Importantly, the age-stratified analysis suggested lower first-attempt success in patients older than 65 years; certainty was low, techniques were heterogeneous, and ultrasound was not consistently used from the initial attempt (13).

A trial restricted to older surgical patients and using the same ultrasound-guided technique, catheter, and outcome definitions in both groups is therefore needed to isolate the effect of access site. We hypothesize that ultrasound-guided distal radial artery catheterization is noninferior to conventional radial artery catheterization for first-attempt success. We further hypothesize that distal access will improve waveform stability and reduce catheter-related problems and movement-related discomfort, although it may require a longer insertion time.

## Materials and methods

### Study design and reporting framework

This investigator-initiated study is a single-center, prospective, two-group, randomized, controlled, noninferiority trial with a 1:1 allocation. The protocol was prepared in accordance with the SPIRIT 2025 statement (14). A completed SPIRIT 2025 checklist will accompany the journal submission. The schedule of enrollment, interventions, and assessments is presented in Figure 1, and the planned participant flow through recruitment, randomization, follow-up, and analysis is shown in Figure 2.

**Figure 1:**
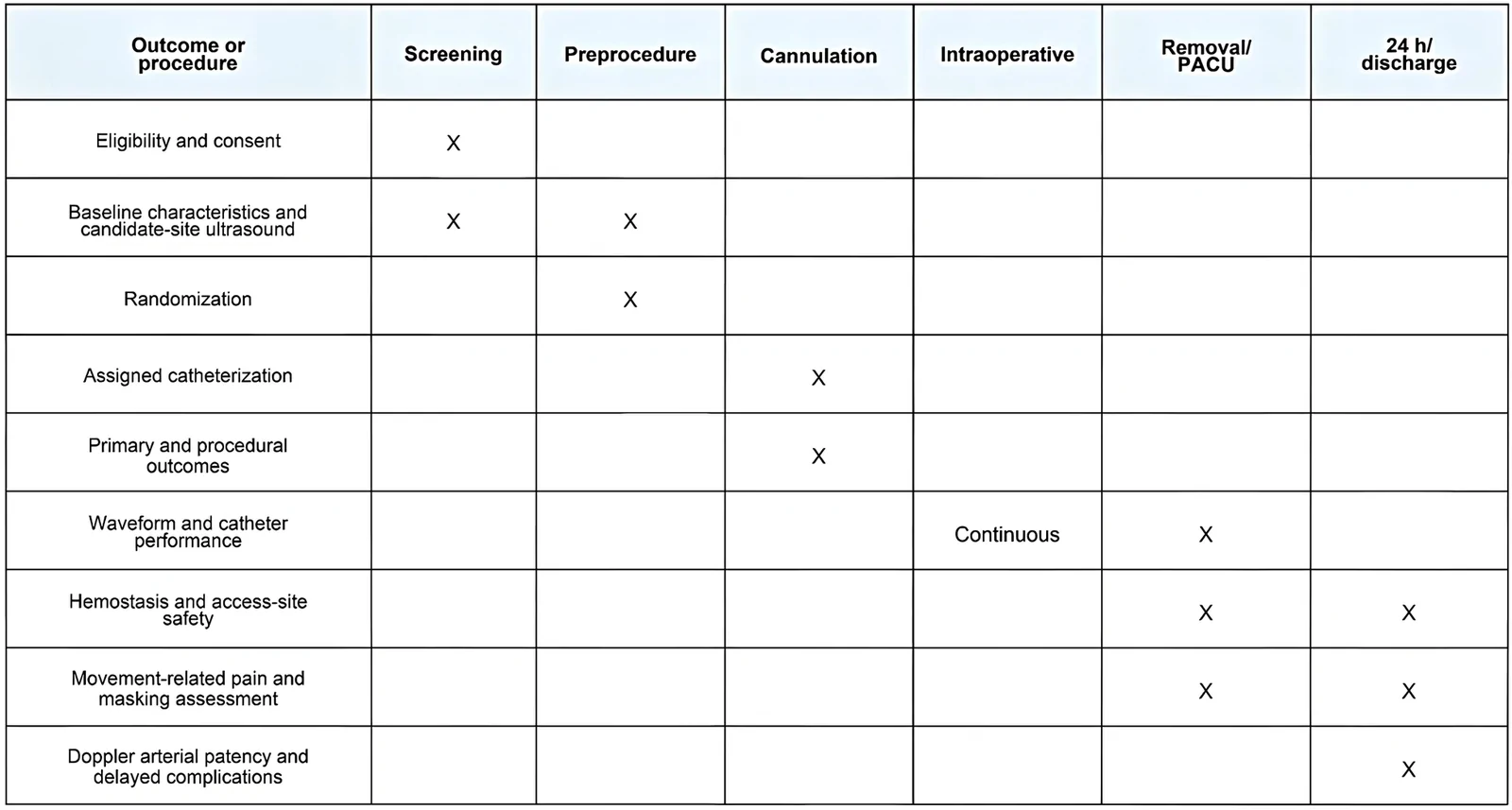
Schedule of enrollment, interventions, and assessments.

**Figure 2:**
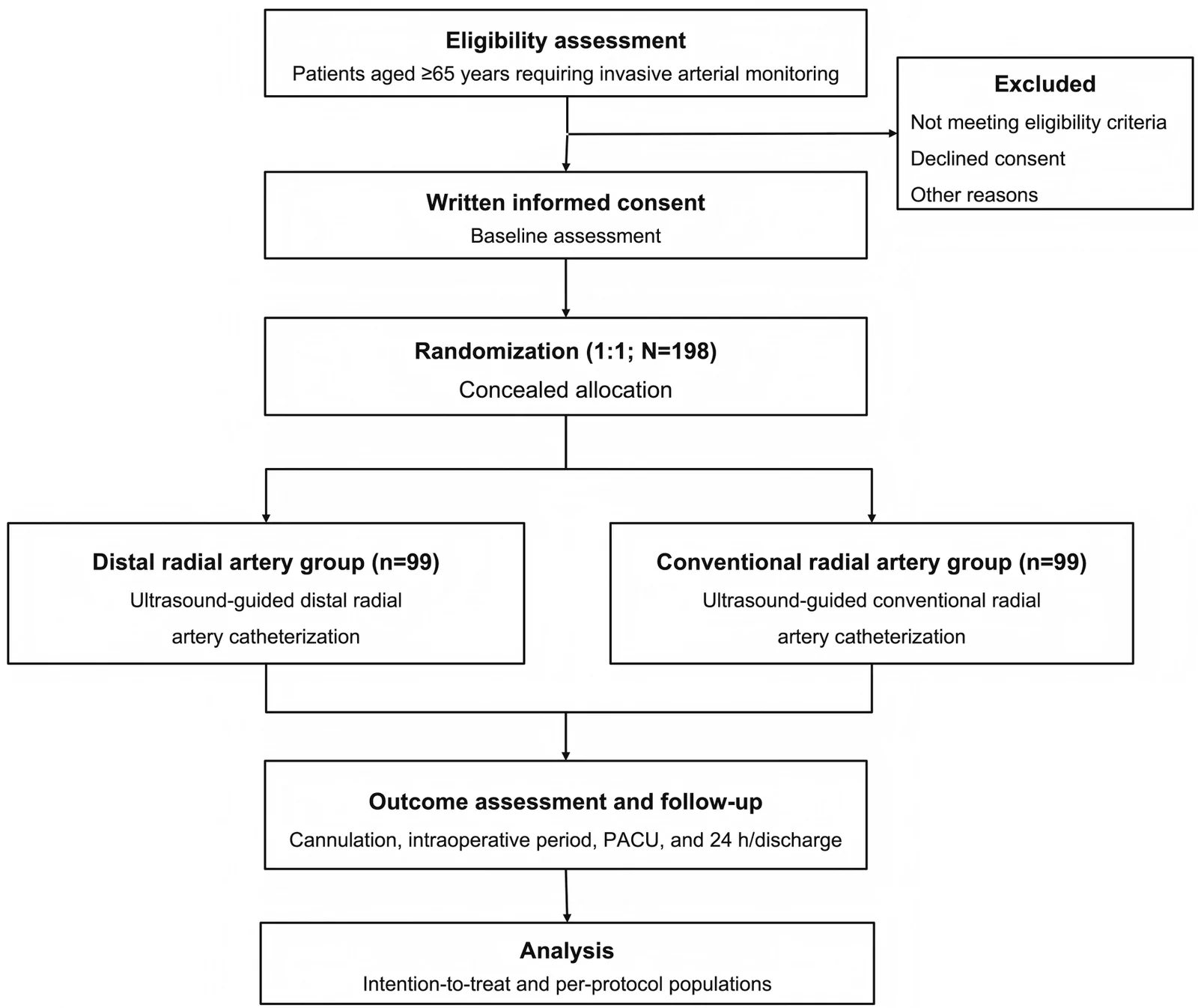
Planned participant flow through recruitment, randomization, follow-up, and analysis.

### Study setting and timeline

The trial will be conducted in the operating rooms of Ningbo No. 6 Hospital, Ningbo, Zhejiang, China. Participants will be screened among patients scheduled for surgery for whom the attending anesthesiologist has independently determined that invasive arterial pressure monitoring is clinically indicated. Study participation will not create an indication for arterial catheterization.

Planned preparatory activities, including ethics approval, prospective registration, database construction, and operator training, will be completed before recruitment. The study is currently in the subject recruitment phase, with the first subject enrolled on July 26,2026. Subject enrollment is expected to be completed by June 2027, followed by database cleansing and analysis between July and August 2027, and manuscript preparation between September 2027 and February 2028. These dates will be updated to reflect the actual approval and recruitment dates before submission.

### Eligibility criteria

#### Inclusion criteria

Participants must meet all of the following criteria:

1. Age 65 years or older.
2. Scheduled for surgery at the study hospital.
3. Invasive radial arterial pressure monitoring was considered clinically indicated by the attending anesthesiologist.
4. A palpable or ultrasound-identifiable distal and conventional radial artery in at least one upper limb.
5. Capacity to understand the study and provide written informed consent, or availability of a legally authorized representative when permitted by the Ethics Committee.

#### Exclusion criteria

Participants will be excluded for any of the following:

1. Infection, burn, major trauma, or skin breakdown at either candidate puncture site.
2. Inadequate collateral hand perfusion, defined as failure of palmar reperfusion within 15 s during a modified Allen test or absent ulnar or palmar-arch flow on Doppler ultrasonography.
3. Severe uncorrected coagulopathy or thrombocytopenia judged to create an unacceptable puncture risk.
4. Existing ipsilateral arteriovenous fistula, previous radial artery harvest, known radial artery occlusion, or major surgery involving the candidate artery.
5. Severe upper-limb deformity or contracture that prevents standardized positioning.
6. Emergency surgery in which consent and study procedures would delay care.
7. Previous enrollment in this trial.

### Recruitment and informed consent

A trained investigator will screen the elective operating list and confirm eligibility with the attending anesthesiologist. Potential participants will receive written and verbal information in understandable language and will have adequate opportunity to ask questions. Written informed consent will be obtained before randomization. Refusal or withdrawal will not affect clinical care. Participants may withdraw permission for future data collection at any time; data already collected before withdrawal will be retained unless the participant requests otherwise and applicable law permits deletion.

### Randomization and allocation concealment

An independent statistician who is not involved in recruitment, catheterization, outcome assessment, or analysis will generate a 1:1 allocation sequence using randomly varying block sizes. Stratification will be by planned operative position (supine vs. non-supine) because positioning may influence catheter stability and wrist movement. The sequence will be transferred to sequentially numbered, opaque, tamper-evident, sealed envelopes by a person independent of enrollment.

After eligibility confirmation, consent, and baseline assessment of both candidate access sites, the research coordinator will open the next envelope immediately before catheterization and inform only the operator. The envelope number, date, participant identifier, and operator will be recorded. Premature opening, a damaged envelope, or deviation from sequence will be documented as an allocation-concealment deviation.

### Masking

The access site makes operator masking impossible. Participants will be shielded from viewing the arm during catheterization, and both sites will be covered with standardized opaque dressings; nevertheless, complete participant masking cannot be guaranteed because the sites are anatomically distinct. At the first postoperative assessment, before site-specific examination or dressing removal, participants will be asked which group they believe they received and how confident they are; responses will be reported descriptively.

The independent postoperative assessor will not participate in allocation or catheterization and will remain unaware of group assignment until site-specific examination or dressing removal, after which masking may be compromised. Standardized criteria will therefore be used for all clinical assessments. Doppler images will be labeled with coded identifiers and interpreted by an assessor unaware of allocation whenever feasible. The statistician will receive groups coded as A and B and remain masked until the primary intention-to-treat (ITT) and per-protocol (PP) analyses are finalized.

Immediate procedural outcomes cannot be fully assessor-masked. They will therefore be recorded by a trained observer who is independent of the operator, using prespecified definitions and a synchronized digital timer. Monitor screenshots or electronic timestamps confirming the first valid arterial waveform will be retained for audit.

### Operator qualification and run-in training

Catheterizations will be performed by a prespecified group of senior anesthesiologists who have completed standardized training in both access sites and the study ultrasound technique. Before enrolling participants, each operator must demonstrate protocol adherence in simulation and complete at least 20 successful supervised distal and 20 successful conventional ultrasound-guided radial catheterizations, or provide documented equivalent recent experience. Operator identity and cumulative study case number will be recorded to assess clustering and learning effects.

### Standardized preprocedural assessment

Standard monitoring will be applied before arterial catheterization. The side of catheterization will be selected according to surgical requirements, the noninvasive blood pressure cuff, prior vascular procedures, and patient-specific contraindications; the side will be recorded before group allocation whenever possible.

Before allocation disclosure, both candidate sites in the selected limb will be examined in short and long axes with a high-frequency linear-array transducer, without excessive probe pressure. At each site, anteroposterior internal diameter, depth from the skin to the anterior arterial wall, visible calcification or plaque, and color-Doppler flow will be recorded. Diameter and depth will be measured at end diastole and averaged across three images. Baseline patency of both the distal and conventional radial arteries will be confirmed.

No sedative will be administered solely for catheterization before the determination of the primary outcome. If clinically indicated, anxiolysis or induction of anesthesia occurs first, the drug and timing will be recorded. Unless contraindicated, 0.5 mL of 1% lidocaine will be infiltrated subcutaneously with a fine needle while avoiding compression or distortion of the target artery.

### Intervention procedures

The same ultrasound platform (FUJIFILM Sonosite, Seattle, Washington, USA), high-frequency linear-array transducer, sterile preparation, 20G*45 mm polyurethane over-the-needle arterial catheter (Shandong, China), flush system, and dynamic needle-tip positioning method will be used in both groups. The artery will be visualized in a short-axis out-of-plane view. The operator will advance the needle in small increments while alternately moving the transducer to keep the needle tip visible within the center of the lumen. After sufficient intraluminal advancement, the catheter will be threaded, connected to a pressurized flush system and a zeroed transducer, and secured with a standardized transparent dressing. Correct placement requires free aspiration and an undamped arterial waveform.

#### Distal radial artery group

All puncture procedures in this study will be performed by attending anesthesiologists with more than 10 years of clinical experience in radial artery catheterization, who have completed standardized training for the distal radial artery puncture technique at the Hegu point prior to study initiation. The forearm will be pronated with the hand resting comfortably, and the thumb neutral or slightly abducted. The distal radial artery will be identified within a prespecified distal zone at Hegu point region (12). The puncture point will be selected where ultrasound shows the most favorable combination of lumen diameter, depth, and straight course; the exact distal subsite will be recorded. If the arterial diameter is <1.5 mm, the procedure is deemed unsafe; no puncture will be performed, and the procedure will be directly classified as a failure.

#### Conventional radial artery group

The forearm will be supinated, and the wrist extended approximately 30 degrees with a soft roll, avoiding excessive extension that may flatten the vessel. The conventional radial artery will be identified 1–2 cm proximal to the wrist crease, lateral to the flexor carpi radialis tendon. The puncture point will be selected where ultrasound shows the most favorable combination of lumen diameter, depth, and straight course within the prespecified conventional zone.

### Definition of attempts, failure, and rescue

An attempt begins when the study catheter needle penetrates the skin and ends when the catheter is successfully placed or the needle is completely withdrawn from the skin. Needle redirection without complete withdrawal will be counted separately. First-attempt success is defined as successful catheterization completed after a single skin puncture. Fine adjustments in the intradermal and intravascular directions are permitted until the catheterization procedure is successfully completed. If catheterization fails or re-puncture is required, this is considered a failure of the first attempt (15). Overall assigned-site success refers to successful catheter placement at the pre-specified assigned site after multiple puncture attempts, with fewer than three skin punctures performed, or within 5 min; eventual successful arterial monitoring after rescue refers to successful monitoring after switching to the rescue puncture site when the assigned site puncture fails (15). Detailed information regarding the rescue procedure shall be documented. Rescue success will not change the assigned-site outcome, and participants will remain in their randomized group for ITT analysis. All cases with missing primary endpoint data will be defined as failure, regardless of which intervention group they belong to.

Reasons for discontinuing an attempt include expanding hematoma, suspected arterial injury, severe vasospasm, new neurologic symptoms, clinically important bleeding, hemodynamic deterioration requiring immediate treatment, or participant request. Clinical stabilization always takes priority over research procedures.

### Catheter maintenance and removal

After placement, the transducer will be leveled at the phlebostatic axis and zeroed according to institutional practice. A square-wave flush test will be performed to identify over- or under-damping. Catheter fixation, extension tubing, pressure bag, flush solution, and monitoring system will be standardized. Wrist splints will not be routinely used; any splint use and reason will be recorded.

The arterial catheter will remain in place only while clinically indicated and will be removed according to routine care by staff unaware of the study hypotheses when feasible. Catheter dwell time, unplanned removal, reinsertion, inability to aspirate blood, blockage, kinking, disconnection, and any period of unusable waveform will be recorded. After removal, standardized manual compression will be applied, and the time to complete hemostasis will be measured.

### Outcomes

#### Primary outcome

The primary outcome is first-attempt catheterization success at the randomized site. Success requires advancement of the catheter into the artery, free blood aspiration, and acquisition of an undamped arterial pressure waveform after one skin puncture.

#### Secondary outcomes

The prespecified secondary outcomes are:

1. Overall catheterization success at the randomized site within three skin punctures and 5 min.
2. Procedural efficiency, assessed by the number of skin punctures, the number of needle redirections, and the needle-to-waveform time among participants with assigned-site success.
3. Diameter of the radial artery at the puncture site.
4. Intraoperative catheter performance, defined primarily as at least one episode of damping or waveform loss requiring manipulation, flushing, limb repositioning, or catheter replacement; episode count and causes will also be recorded.
5. Access-site safety and hemostatic status should be assessed 24 h post-puncture, or immediately prior to discharge if the patient is discharged earlier than this 24-hour time point (the same applies hereafter), including hematoma, persistent bleeding, clinically suspected vasospasm, symptomatic nerve injury, pseudoaneurysm, infection, clinically important hand ischemia, and time to complete hemostasis after catheter removal. Researchers will conduct a structured telephone interview with all enrolled patients 7 days after the procedure, to inquire about and record any symptoms related to late complications including local redness, swelling, persistent pain, abnormal local masses, paresthesia and other discomforts. Due to limitations in sample size, this section contains only exploratory results.
6. Patency of the distal and conventional radial arteries on color and pulsed-wave Doppler at 24 h. If the study catheter remains *in situ*, patency will additionally be assessed within 2 h after removal when feasible. The specific procedure involves fully exposing the punctured segment of the radial artery, positioning the high-frequency ultrasound probe perpendicular to the long axis of the artery to obtain the maximum transverse cross-sectional diameter of the radial artery. To assess radial artery patency, the probe is slowly slid along the long axis of the radial artery from the distal puncture point toward the proximal forearm to confirm the continuity of blood flow signals; if no continuous blood flow signals are detected within the lumen, an auxiliary compression test is performed to confirm occlusion, and the precise extent of the affected segment is meticulously documented. Vascular spasm is defined as the presence of a lumen with reduced caliber, accompanied by slowed blood flow that does not completely cease. Low flow is defined as detectable blood flow with a velocity below the normal threshold. True occlusion is defined as the absence of blood flow signals within the lumen and the absence of distal pulsation. All examination findings are stored as imaging data to facilitate offline verification when necessary (16, 17). Occlusion is defined as the absence of both color-flow and pulsed-wave Doppler signals in the relevant segment.

#### Exploratory and descriptive measures

Exploratory measures are first-pass success without needle withdrawal or redirection; total procedure time from sterile probe contact to the first valid waveform; posterior-wall puncture observed on ultrasound; failure to obtain an arterial blood sample when clinically requested; and movement-related puncture-site pain on a 0–10 numerical rating scale at the first postoperative assessment and at 24 h or discharge. Catheter dwell time, blockage, kinking, disconnection, dislodgement, unplanned removal, and reinsertion will be described as exposure or malfunction variables. Ultrasound-measured diameter, depth, calcification, or plaque, and baseline flow are anatomical covariates rather than outcomes. Rare major vascular events will be summarized descriptively because the trial is not powered for them.

### Outcome definitions and assessment schedule

Outcome assessors will use a prespecified manual. Complete hemostasis is the first time no visible bleeding recurs for 30 s after release of manual compression. Hematoma will be measured at its greatest diameter and categorized as less than 2 cm, 2–5 cm, or greater than 5 cm. Clinically important hand ischemia requires a new perfusion abnormality that prompts catheter removal, vascular imaging, or specialist assessment. Suspected arterial occlusion must be confirmed by absent color-flow and pulsed-wave Doppler signals. The timing of every assessment and any deviation from its prespecified window will be recorded.

### Baseline and perioperative variables

Baseline data will include age, sex, body mass index, ASA physical status, smoking, diabetes, hypertension, coronary or peripheral arterial disease, chronic kidney disease, antiplatelet or anticoagulant therapy, and relevant preoperative laboratory results obtained as part of routine care. Perioperative data will include procedure type, anesthetic technique, operative position, pre-cannulation arterial pressure, vasopressor use before cannulation, catheter side, operator, timing relative to induction, operative duration, catheter dwell time, and clinically relevant changes in hand perfusion.

### Sample size

The sample size is based on the primary noninferiority hypothesis. The first-attempt success rate in the conventional group is assumed to be 85%, informed by prior studies in older or high-risk patients and contemporary comparative trials (6, 8, 10). We assume an equal 85% success rate in the distal group. The noninferiority margin is 15 percentage points on the absolute risk-difference scale. This margin was selected before recruitment and represents the largest loss in first-attempt success considered clinically acceptable in exchange for the anticipated benefits of improved waveform stability, reduced wrist-motion interference, easier hemostasis, and preservation of the proximal radial artery; the same margin has been used in the prior perioperative noninferiority trials (8, 18).

With a one-sided alpha of 0.025% and 80% power, 89 evaluable participants per group are required. Allowing for 10% attrition, withdrawal, or major protocol deviation yields 99 participants per group (198 total). The calculation was performed with PASS version 15.0 (NCSS, Kaysville, Utah, USA). Because the primary outcome is measured immediately, true missingness is expected to be minimal. Recruitment will not stop early for apparent benefit or futility. Lastly, this study will adopt a 10% noninferiority margin to perform sensitivity analyses and exploratory interpretations, to further clarify the clinical value of the study findings.

### Statistical analysis

A detailed statistical analysis plan will be finalized, dated, and made publicly available before database lock and unmasking of group codes. Analyses will be performed using R (R Foundation for Statistical Computing, Vienna, Austria) and SPSS version 26.0 (IBM, Armonk, New York, USA). Secondary and exploratory analyses will emphasize effect estimates with 95% confidence intervals. Because no multiplicity adjustment or confirmatory hierarchy is planned, *P* values for these analyses will be interpreted as descriptive and hypothesis-generating rather than as proof of additional treatment effects.

### Analysis populations

The ITT population will include all randomized participants in their assigned group, regardless of the site ultimately used or protocol deviations. The PP population will exclude participants with major prespecified deviations that could affect the primary comparison, including use of a nonassigned initial site, non-ultrasound initial technique, nonstudy catheter, ineligible enrollment, or failure to initiate the allocated procedure for a nonclinical reason (19). Safety analyses will be based on the site at which the initial study puncture was actually performed, with a sensitivity analysis by randomized group.

Protocol deviations are defined as cases that do not meet the inclusion criteria but are mistakenly enrolled, or cases that meet the exclusion criteria after enrollment; crossover is defined as patients randomized to one group but receiving the intervention of the other group; rescue catheterization is defined as cases where the initial puncture fails and another puncture site of the opposite group is selected for re-puncture; the criteria for excluding patients from the per-protocol population also include major deviations from inclusion or exclusion criteria, failure to complete the intervention as planned, and missing data for the primary endpoint. All these definitions have been formulated and added to the study protocol in advance.

### Primary analysis

The effect measure will be the absolute risk difference in first-attempt success (distal minus conventional), with a two-sided 95% Miettinen-Nurminen score confidence interval for two independent proportions. Distal catheterization will be declared noninferior only if the lower confidence limit is greater than −0.15 in both the ITT and PP populations, in keeping with the recommended interpretation of noninferiority trials (20). If noninferiority is established, superiority will be explored only if the same confidence interval excludes zero in favor of distal access.

For the ITT primary analysis, any participant with an unobserved primary outcome will be handled conservatively as a failure in the distal group and a success in the conventional group. Sensitivity analyses will include complete-case analysis, best-worst and worst-best bounds, and multiple imputation when missingness is sufficient to support a model.

### Secondary analyses

Secondary outcomes will be summarized by randomized group with effect estimates and 95% confidence intervals. Dichotomous outcomes will be reported as risk differences and risk ratios. Continuous outcomes will be summarized as the mean (standard deviation) or the median (interquartile range) and analyzed with regression or rank-based methods appropriate to their distributions. Attempt counts will use Poisson or negative-binomial regression after assessment of dispersion. For composite catheter-performance and safety outcomes, the composite and clinically important components will both be reported.

Exploratory pain scores will be summarized by assessment time and compared using rank-based or regression methods appropriate to their distribution. Because these and other exploratory analyses are not adjusted for multiplicity, they will be interpreted as hypothesis-generating.

A supportive adjusted analysis of the primary outcome will use logistic regression with prespecified prognostic variables: age, pre-cannulation mean arterial pressure, assigned-site arterial diameter, visible calcification or plaque, and operator. Adjusted risk differences will be obtained by marginal standardization with 95% confidence intervals. The operator will be included as a fixed effect when the number of operators is small; otherwise, cluster-robust standard errors will be used.

### Prespecified subgroup analyses

Exploratory interaction analyses will assess treatment-effect modification by age and assigned-site arterial diameter, modeled as continuous variables where functional form permits. Results will be reported as interaction estimates and confidence intervals rather than within-subgroup *P* values. These analyses are not powered for definitive inference.

### Data collection and management

Data will be entered into a password-protected electronic case-report form using coded participant identifiers. The linkage file containing personal identifiers will be stored separately on a restricted hospital server. Range checks, required fields, programmed logic checks, and time-stamped audit trails will be used. Two trained researchers will independently verify consent, eligibility, randomization, the primary outcome, and serious adverse events. Discrepancies will be resolved against source records by a senior investigator who was not the original data entrant.

Only authorized study personnel will have role-based access. The database will be locked only after all queries are resolved and the statistical analysis plan is signed. Study records will be retained for the period required by the ethics committee, hospital policy, and applicable regulations. Any protocol amendment affecting eligibility, outcomes, sample size, or analysis will receive ethics approval and registry updating before implementation when practicable.

### Safety monitoring and adverse-event management

Both study procedures are variations of established clinical arterial access. Expected events include transient pain, a small hematoma, bleeding, vasospasm, temporary arterial occlusion, and catheter malfunction. An adverse event will be classified by severity, seriousness, expectedness, relationship to the procedure, action taken, and outcome. Serious adverse events include events resulting in death, life-threatening deterioration, prolonged hospitalization, persistent disability, urgent vascular or surgical intervention, or another medically important condition.

Hand color, temperature, capillary refill, pulse oximetry waveform on an ipsilateral finger, sensory symptoms, motor function, and Doppler flow will be assessed promptly if vascular compromise is suspected. The catheter will be removed, and pressure released or reapplied as clinically appropriate. Persistent ischemia, expanding hematoma, suspected compartment syndrome, pseudoaneurysm, or neurologic deficit will trigger immediate senior anesthesiology review and vascular or hand surgery consultation.

Given the modest sample size, short follow-up, familiar procedures, and low expected rate of serious events, a formal independent data monitoring committee is not planned. Safety will instead be reviewed after the first 20 participants and then every 50 participants by an independent senior clinician who is not involved in catheterization. Recruitment will be suspended for any unexpected procedure-related serious adverse event until causality and necessary protocol changes are reviewed.

### Quality control

Before recruitment, all staff will be trained in eligibility assessment, consent, allocation concealment, the ultrasound technique, outcome definitions, Doppler patency assessment, and adverse-event reporting. A procedural checklist will be completed for every catheterization. Ultrasound settings and measurement methods will be standardized, and devices will undergo routine calibration and maintenance.

The coordinating investigator will audit the first five procedures by each operator and a random 10% sample thereafter. Audit items will include envelope integrity, assigned site, catheter type, ultrasound method, timing, attempt counts, waveform documentation, and follow-up completeness. Inter-rater agreement for Doppler patency and clinical hematoma assessment will be evaluated in a training sample before recruitment.

### Patient and public involvement

Patients and members of the public were not involved in the initial design of this protocol. Before recruitment, the participant information sheet and wording of the pain assessment will be reviewed by two older patient representatives for clarity and burden. Their comments and resulting changes will be documented. Participants may request a plain-language summary of the results.

## Ethics and dissemination

### Ethics approval and consent to participate

The protocol, informed-consent form, case-report form, recruitment materials, and any amendments must receive written approval from the responsible ethics committee before enrollment. The trial will be prospectively registered before the first participant is randomized. It will be conducted in accordance with the 2024 revision of the World Medical Association Declaration of Helsinki (21) and applicable Chinese regulations. The approved protocol will be supplied as supporting information at journal submission, and all bracketed ethics and registration fields must be replaced with verified information.

### Confidentiality

Study reports will contain no direct identifiers. Publications and shared datasets will use de-identified or appropriately anonymized data. Access to identifiable source documents will be limited to authorized investigators, monitors, the ethics committee, and regulatory authorities as permitted by law.

### Dissemination and data sharing

Results will be submitted to a peer-reviewed journal and presented at relevant scientific meetings regardless of whether the primary hypothesis is supported. Authorship will follow the International Committee of Medical Journal Editors criteria. After publication, the investigators plan to share the protocol, statistical analysis plan, data dictionary, analysis code, and a de-identified participant-level dataset in a recognized repository, subject to participant consent, ethics approval, and privacy requirements. Any restrictions and the process for controlled access will be stated transparently.

## Discussion

This trial will test whether ultrasound-guided distal radial artery catheterization preserves clinically acceptable first-attempt success in older surgical patients when the access site is the principal difference between groups. Distal access may improve waveform stability during wrist movement, facilitate compression, and preserve the conventional forearm segment, but these advantages would not justify a clinically important increase in failed punctures, delayed monitoring, or tissue trauma.

It should be further noted that variations in the puncture site can exert subtle effects on the puncture procedure protocol, encompassing multiple aspects such as hand positioning, ultrasonic probe placement, and puncture fixation techniques. The primary focus of this study is not merely the differences in puncture sites, but rather the complete procedural protocols corresponding to different puncture sites.

Previous trials have produced inconsistent estimates of technical success. Xiong et al. found noninferior first-attempt success and fewer dampened waveforms with distal access, whereas Maitra et al. reported lower first-attempt success and longer catheterization (8, 9). More recent studies found comparable insertion success but fewer waveform-loss events, and in one trial, shorter hemostasis, with distal placement (10, 11). A meta-analysis of 12 randomized trials found no overall difference in first-attempt success and suggested fewer local complications and catheter-performance problems but longer insertion time; its low-certainty subgroup analysis suggested reduced first-attempt success among patients older than 65 years (13). Technique, arterial size, ultrasound use, operator experience, and outcome definitions varied across studies.

The present design addresses several sources of heterogeneity by measuring both candidate sites before allocation disclosure, using ultrasound from the first attempt in both groups, standardizing the catheter and dynamic needle-tip technique, and recording operator case sequence. The noninferiority framework is appropriate because distal access is expected to offer secondary advantages rather than superior immediate technical success. Nevertheless, the 15-percentage-point margin, although used in a previous perioperative trial (8), is clinically generous. The absolute risk difference and confidence interval will therefore be interpreted alongside waveform performance, hemostasis, patency, and access-site harm; noninferiority alone will not establish distal access as the default approach.

Important limitations remain. Operators cannot be masked, and the primary outcome is operator-dependent. Training, an independent observer, electronic waveform confirmation, and protocol audits can reduce but not eliminate performance and ascertainment bias. The relatively lenient non-inferiority margin (15%) may result in an insufficient sample size for this study, thereby reducing the statistical power to test certain secondary outcomes. Postoperative masking may be compromised after dressing removal. The single-center setting and experienced operators may limit generalizability. Follow-up for 24 h is appropriate for early catheter failure, hematoma, and occlusion, but may miss delayed thrombosis, infection, pseudoaneurysm, or neurologic symptoms. The trial is not powered for rare events or superiority on secondary outcomes. Due to the limitations of the sample size, the safety outcomes only include exploratory analyses.

The most useful result may therefore be a conditional recommendation. If distal access is noninferior and improves waveform continuity or hemostasis, it may be favored when wrist movement, operative positioning, or preservation of the conventional radial segment matters. If noninferiority is not established, anatomical and operator data may still identify patients with an adequate distal diameter and favorable vessel course in whom the technique remains reasonable. Either result should inform operator training and a subsequent multicenter effectiveness trial.

## Trial status

Protocol version 1.0 is dated 1st March 2026. Recruitment must not begin until ethics approval and prospective trial registration are complete. The actual recruitment start date, current recruitment status, and anticipated completion date will be updated immediately before journal submission.
